# Serological and Molecular Characterization of Prevalent Olive-Associated Viruses in Saudi Arabia

**DOI:** 10.3390/v18030328

**Published:** 2026-03-06

**Authors:** Mahmoud A. Amer, Muhammad Amir, Khadim Hussain, Ibrahim M. Al-Shahwan, Mohammed A. Al-Saleh

**Affiliations:** 1 Plant Protection Department, College of Food and Agriculture Sciences, King Saud University, P.O. Box 2460, Riyadh 11451, Saudi Arabia; mamaamery@yahoo.com (M.A.A.); amiraim1122@gmail.com (M.A.); ishahwan@ksu.edu.sa (I.M.A.-S.); 2 Chair of Date Palm Research, Centre for Chemical Ecology and Functional Genomics, College of Food and Agriculture Sciences, King Saud University, Riyadh 11451, Saudi Arabia

**Keywords:** olive, viruses, OEGV, TuYV, OMMV, ArMV, CLRV, TNV-D, SLRSV, PCR

## Abstract

A survey was carried out during 2023–2024, and 363 asymptomatic and symptomatic olive samples with deformed leaves, mosaic, and yellow spots were collected from different regions in Saudi Arabia. These samples were tested by ELISA against eight important olive viruses. To investigate the presence of these viruses in olive trees, PCR and RT-PCR techniques were employed using the virus-specific primers. The obtained results from serological tests indicated that 44.4% of the collected samples were found to be positive with at least one of the tested viruses. The most prevalent virus was OEGV (14.3%), followed by ArMV (11.9%), SLRSV (11.3%), CLRV (9.4%), TuYV (5%), TNV-D (4.4%), OMMV (3.6%), whereas OLV-1, OLV-2, CMV, TMV, OLV-3, OLYaV, and OLRSV were not positive in the tested samples. Single, as well as mixed infections, were observed in a number of samples with 9.4% and 34.7%, respectively. The nucleotide sequence analysis of PCR amplified fragments revealed 99.7–100% identity to OEGV, 95–99% to TuYV, 85–98% to OMMV, 83–93% to ArMV, 92–97% to CLRV, 84–98% to TNV-D, and 85–97% to SLRSV isolates, according to the pairwise nucleotide identity percentage calculated by SDT software. This is the first comprehensive survey to investigate the genetic diversity and phylogenetic relationship of seven olive viruses detected in olive trees in Saudi Arabia, which can provide the missing local epidemiological understanding.

## 1. Introduction

One of the oldest domesticated fruit trees and the most widely grown fruit crop worldwide is the olive tree (*Olea europaea* L.) [1]. In Saudi Arabia, according to the Food and Agriculture Organization’s 2021 statistics, Olive is a major and economically important crop and produces 382 thousand tons of olive fruits annually, ranking ninth globally and fifth in the Arab world out of the top 29 countries. About 2% of the world’s olive production comes from Saudi Arabia [2]. With olive oil accounting for 5.5% of the Kingdom’s total exports and ranking as the fifth-largest export that generates foreign exchange, olive production is essential to the country’s economy [3]. Numerous fungi, bacteria, viruses, phytoplasmas, and other biotic agents of diseases of unknown etiology have been observed to infect olive trees [4,5].

Olive viruses are still not well understood; nonetheless, in contrast to other woody Mediterranean fruit crops like citrus and grapevine. There is little knowledge about viruses that affect olives, even in Spain, which is the world’s biggest producer of olive products [6]. Olive trees are known to be infected by a wide variety of viruses and virus-like diseases [7,8,9,10,11]. These viruses included: Olive latent virus 1 (OLV-1) [12], Olive mild mosaic virus (OMMV) and Tobacco necrosis virus Strain D (TNV-D) as a members of Tombusviridae [13,14,15]; Olive latent ringspot virus (OLRSV) [16,17], Cucumber mosaic virus (CMV) and Olive latent virus 2 (OLV-2) from Bromoviridae [18]; Strawberry latent ring spot virus (SLRSV) [19,20], Arabis mosaic virus (ArMV) [9,20], Cherry leaf roll virus (CLRV) from Secoviridae [11], Tobacco mosaic virus (TMV; Virgaviridae) [21], Olive latent virus 3 (OLV-3; *Tymoviridae*) [22], Olive semilatent virus (OSLV; unassigned) [23], Olive yellow mottling and decline-associated virus (OYMDaV; unassigned) [24], Olive leaf yellowing associated virus (OLYaV) [11,25], Olive vein yellowing-associated virus (OVYaV; *Closteroviridae*) [26]. Olea europaea geminivirus (OEGV; *Geminiviridae*), which can also infect olive plants, was classified as a putative member of a new genus within the *Geminiviridae* family [7,27,28]. One of the most significant viruses in the *Polerovirus* genus (*Solemoviridae* family) is the turnip yellows virus (TuYV) [29,30].

Plant pathogenic viruses belonging to the *Geminiviridae* family are significant because they can cause severe diseases across the world [31,32,33]. The single-stranded, circular DNA genomes of these viruses (2500–5200 nt), which are classified as *Geminiviridae*, are packaged into non-enveloped geminate particles that are differentiated by their host range, vector transmission, and genome organization [34]. The *Geminiviridae*, *Tombusviridae*, and *Secoviridae* viral families are all non-enveloped plant viruses that cause economically significant diseases worldwide, despite differences in their genomic material, replication mechanisms, and fundamental biological traits. Members of the *Tombusviridae* and *Secoviridae* families, on the other hand, contain linear, positive-sense single-stranded RNA genomes and simple icosahedral capsids. Proteins are created by cleaving large polyproteins. *Geminiviridae*, *Tombusviridae*, and *Secoviridae* are families of plant viruses distinguished by key characteristics of their genome and structure [35,36].

The Al Jowf region is one of Saudi Arabia’s most productive agricultural areas, producing wheat, alfalfa, olives, barley, fruits, and vegetables [2]. In Saudi Arabia, no previous studies have been conducted on the olive viruses. Due to the lack of knowledge regarding the genetic variability and geographic distribution of olive viruses in Saudi Arabia. The present study comprehensively reports the prevalence of seven important viruses infecting olive trees in different regions of Saudi Arabia, and their economic importance has been discussed. Also, this study contributes new epidemiological and genomic information, expands our understanding of olive virology to a previously overlooked region, and enhances our understanding of the diversity and distribution of olive-associated viruses worldwide.

## 2. Materials and Methods

### 2.1. Field Surveys and Sample Collection

**Figure 1:**
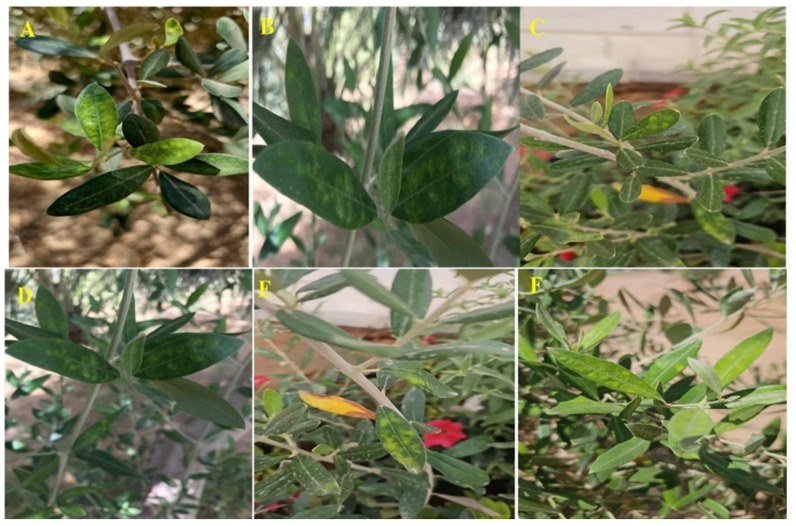
Naturally infected olive tree samples showing leaf mottling symptoms (**A**–**D**), leaf yellowing, chlorosis, and severe yellow discoloration (**E**,**F**).

A field visit was conducted in 2023–2024 in the main olive-producing regions of Saudi Arabia. A total 363 asymptomatic or symptomatic olive trees samples with virus-like symptoms such as mottling, yellow streak leaf, leaf chlorosis, stem fasciation, bifurcation, leaf deformation and chlorotic flecking were collected randomly from 36 different farms (Al Jowf (9 farms), Tabuk (10 farms), Hail (7 farms), Northern Borders (4 farms) and 2 farms from each Qassim, Abha and Al-Bahah regions) (Figure 1).

### 2.2. Serological Detection

Collected samples were tested against the important olive viruses using ELISA against the available eight olive viruses (OLV-1, CLRV, ArMV, SLRSV, TuYV, TNV-D, CMV, and TMV) specific antibody (LOEWE Biochemica, Sauerlach, Germany). The ELISA was performed and analyzed using a BioTek ELx 808 microplate reader (BioTek Instruments, Inc., Winooski, VT, USA) at a wavelength of 405 nm. When these absorbance levels exceeded two times the mean of the relevant negative controls, the ELISA tested samples were considered positive [37].

### 2.3. RT-PCR and PCR Assay

For the detection of olive RNA and DNA viruses, 100 mg of each Phloem tissue sample was utilized for total RNA and DNA isolation using GeneJET Plant RNA and DNA Purification Mini Kit (ThermoFisher Scientific Inc., Vilnius, Lithuania). The One-Step RT-PCR SuperScriptTM IV system (Thermo Fisher Scientific, Waltham, MA, USA) and PCR were performed using specific primers to confirm the presence of the following viruses, including: OMMV, CLRV, ArMV, SLRSV, TNV-D, TuYV, OLV-1, OLV-2, OLV-3, OLRSV, OLYaV, TMV, CMV, as an RNA virus (Table 1) in a Thermal Cycler (Eppendorf Mastercycler Nexus Gradient, Model 6331, Eppendorf SE, Hamburg, Germany), amplifications were performed at 95 °C for 1 min, followed by 35 cycles at 95 °C for 30 s and 54 °C, 58 °C, or 60 °C for 1 min. For ArMV, OLV3, TNV-A, CMV, OLV1, TNV-D, OMMV, and SLRSV, PCR optimizations showed that the optimal annealing temperatures were 54 °C for 1 min, 60 °C for CLRV, OLV2, 58 °C for OLRSV and OLYAV and TMV, and 72 °C for 1 min, followed by 72 °C for 10 min as a final extension step. For OEGV, extracted DNA was amplified using our designed specific primer pairs OEGV-CP-F and OEGV-P-R (Table 1). The following steps were used in the amplification process: 95 °C for 1 min, 35 cycles at 95 °C for 45 s, 60 °C for 1 min, 72 °C for 1 min, and a final extension step of 72 °C for 10 min. For analysis, the PCR product was placed onto a 1% agarose gel and electrophoresed. DNA Ladder (50 bp) HyperLadderTM II (Bioline, London, UK) was used to determine the amplified products.

### 2.4. Nucleotide Sequencing and Phylogenetic Tree Analysis for OEGV

Three samples were selected that were positive for the OEGV as a DNA virus, three isolates that were positive for OMMV, two samples that were positive for the CLRV, and one sample that was positive for each of the ArMV, SLRSV, TuYV, and TNV-D, representative of all the visited regions and locations, were purified using the QIAquick Gel Extraction Kit (Qiagen, Hilden, Germany). These amplified products were sent to Macrogen Inc., Seoul, South Korea, for two-directional Sanger sequencing of the DNA. Sequencing data was aligned using the DNA Star computer software (version 5.0) and the National Center for Biotechnology Information (NCBI). The BLAST tool (NCBI, https://blast.ncbi.nlm.nih.gov/ accessed 29 December 2025) was used to evaluate the sequencing results. Sequence Demarcation Tool (SDT v1.2) and MegAlign (v5.0), as implemented by Lasergene, were used for pairwise sequence comparisons [44]. Sequences aligned with the ClustalW algorithm were used to construct phylogenetic trees using MEGA-X using the Maximum-Likelihood approach with 1000 bootstrap replications as implemented by MEGA 7.0. The Saudi isolates’ nucleotide identity table was created using DNA Star software (version 5.0) and compared to other isolates that were reported in the GenBank database.

## 3. Results

### 3.1. Field Observation

Composite olive samples were collected from different fields in olive in main olive-growing areas in different regions of Saudi Arabia. Symptomatic leaf samples exhibited mild mottling, yellow spots, stem fasciation, bifurcation, Leaf chlorosis, deformation, and chlorotic flecking symptoms (Figure 1).

### 3.2. Detection of the Important Olive Viruses Using ELISA, PCR, and RT-PCR

**Figure 2:**
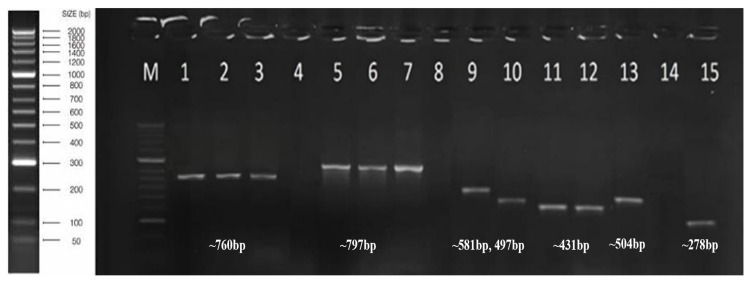
Agarose gel (1%) electrophoreses show selected olive-positive samples using specific primer primers for OEGV (lanes 1, 2, 3) at the exact size ~760 bp, OMMV (lanes 5, 6, 7) at the exact size ~797 bp, TuYV (lane 9) at the exact size ~581 bp, SLRSV (lane 10) at the exact size ~497 bp, CLRV (lanes 11, 12) at the exact size ~431 bp, ArMV (lane 13) at the exact size ~504 bp and TNV-D (lane 15) at the exact size ~278 bp. No results were obtained with the negative control (lanes 4, 8). Lane M1 represents HyperLadderTM II DNA Ladder (Bioline).

Based on the results of the serological test (ELISA), five viruses were detected in olive samples that were collected from seven main regions of Saudi Arabia. These viruses include CLRV, ArMV, SLRSV, TNV-D, and TuYV. The comprehensive analysis of these samples revealed that the number of single viral infections was much lower than the mixed infection with at least two viruses. OEGV and OMMV were detected with the exact size of 760 bp and 797 bp using PCR and RT-PCR from Al-Jowf, Tabuk, and Hail, respectively. Olive-positive samples utilized by ELISA for TuYV, SLRSV, ArMV, and TNV-D were confirmed using RT-PCR with the exact size 581 bp, 497 bp, 504 bp, and 278 bp, respectively, from the Al Jowf region, whereas CLRV was detected with the exact size 431 bp from Al Jowf and Hail regions, as well as Agarose gel (1%) electrophoreses demonstrate olive-positive samples. HyperLadderTM II DNA Ladder (Bioline) was used to determine the amplified products, as shown by agarose gel (1%) electrophoresis (Figure 2). The obtained results based on serological and molecular tests indicated that 44.4% of the collected samples were found to be positive with at least one of the tested viruses. OEGV was the most frequent virus detected, followed by ArMV, SLRSV, CLRV, TuYV, TNV-D, and OMMV, whereas OLV-1, OLV-2, OLV-3, TMV, CMV, OLYaV, and OLRSV were negative in all tested samples (Table 2). The percentage of olive infected samples in each region in Saudi Arabia showed with single infection (9.4%) and mixed infection (34.7%) were detected in most of the tested samples (Table 3 and Table 4) due to the lack of efficient natural barriers to virus accumulation, the use of uncertified planting material, and the vegetative propagation of olives infected with the virus.

### 3.3. Nucleotide Sequence and Phylogenetic Tree for OEGV

Isolates of OEGV, OMMV, TuYV, SLRSV, ArMV, TNV-D, and CLRV with expected size of amplified product of 760 bp, 797 bp, 581 bp, 497 bp, 504 bp, 278 bp, and 431 bp were sequenced using specific primers. These isolates were submitted in the NCBI database with the following accession numbers: PP273426, PP273428, PP273430 for OEGV-SA1, OEGV-SA2, OEGV-SA3 isolates; PX693988, PX693989, PX693990 for OMMV-SA1, OMMV-SA2, OMMV-SA3 isolates; PX693991 for ArMV-SA1 isolate; PX693992 for TNV-D-SA1 isolate; PX693993 for TuYV-SA1 isolate; PX693994 for SLRSV-SA1 isolate; PX693995, PX693996 for CLRV-SA1 and CLRV-SA1 isolates, respectively.

The phylogenetic trees were constructed using the amplified conserved partial genomic sequences of these viruses, e.g., 760 bp sequence of CP for OEGV, 797 bp sequence of CP gene for OMMV, 581 bp sequence of CP gene for TuYV, 497 bp sequence of Polyprotein gene for SLRSV, 504 bp sequence of CP for ArMV, 278 bp sequence of 22 kDa protein gene for TNV-D, and 431 bp sequence of 3′UTR region for CLRV. Phylogenetic tree analysis based on these partial sequences showed that the Saudi isolates for each detected virus are in the same clade as the other isolates that were published from GenBank. Our isolates are completely comparable to one another, according to nucleotide percentage identity. The nucleotide sequence analysis revealed 83–93% to ArMV and 85–98% identity to OMMV, 95–99% to TuYV, 92–97% to CLRV, 84–98% to TNV-D, and 85–97% to SLRSV and 99.7–100% identity to OEGV isolates, according to a distance matrix that displayed the pairwise nucleotide identity percentage in relation to other most similar sequences.

**Figure 3:**
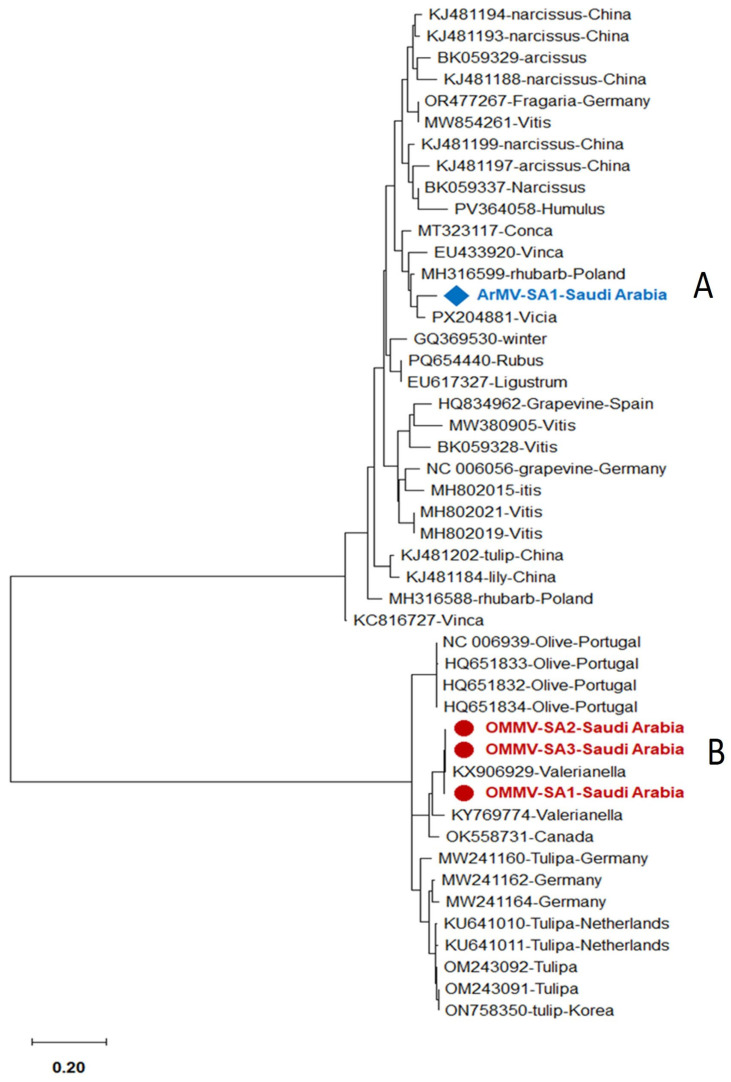
Phylogenetic tree using MEGA-X using the Maximum-Likelihood approach with 1000 bootstrap replications as implemented by MEGA 7.0, based on the alignment of one ArMV (**A**) and three OMMV Saudi Arabian isolates (**B**) sequence with closely related sequences.

**Figure 4:**
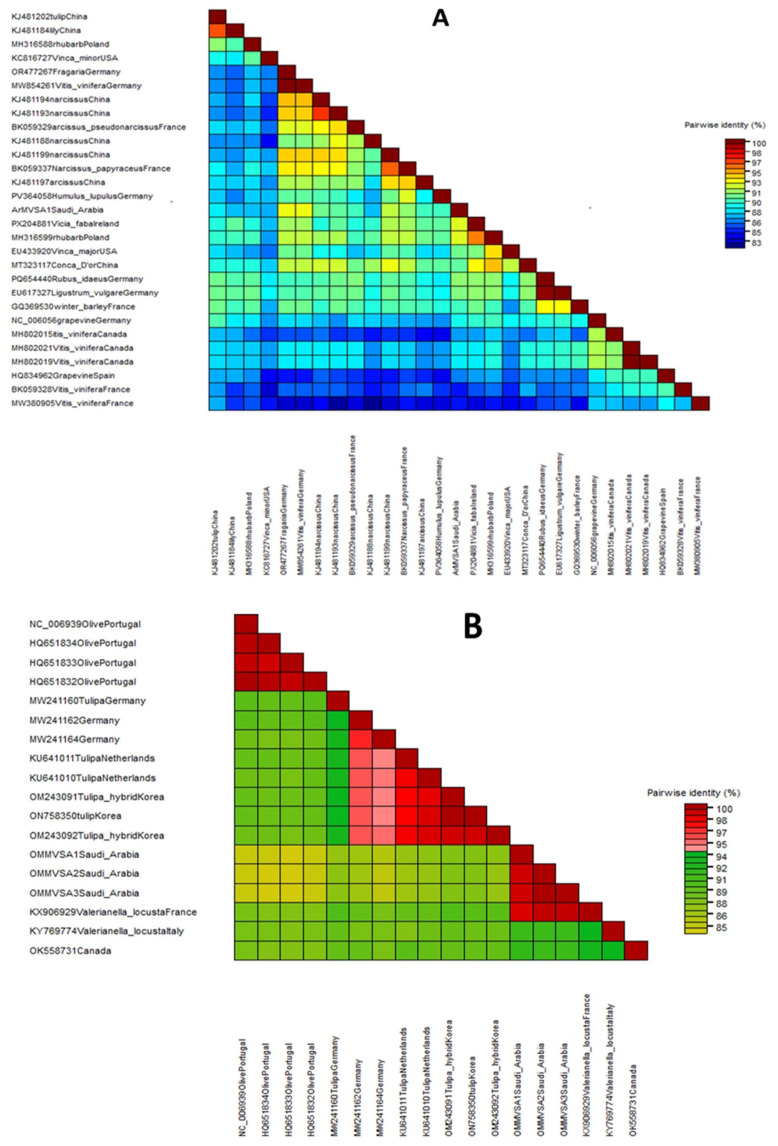
Distance matrix illustrating the pairwise nucleotide identity percentage of one ArMV (**A**) and three OMMV Saudi Arabian isolates (**B**) as compared with other most similar sequences.

The nucleotide sequence percentage identity between ArMV isolates, the highest identity (93%) was observed with two isolates isolated from Fragaria and Vitis vinifera in Germany (OR477267 and MW854261), and the lowest similarity (83%) was observed with two isolates isolated from Vitis vinifera in France (MW380905 and BK059328) (Figure 3 and Figure 4A).

The nucleotide sequence percentage identity between OMMV isolates showed that our isolates are 85–98% similar to each other. The highest identity (98%) was observed with one isolate isolated from Arcissus pseudonarcissus from France (BK059329), and the lowest similarity (85%) was observed with four isolates isolated from olive in Portugal (NC_006939, HQ651832, HQ651834, and HQ651833) (Figure 3 and Figure 4B).

**Figure 5:**
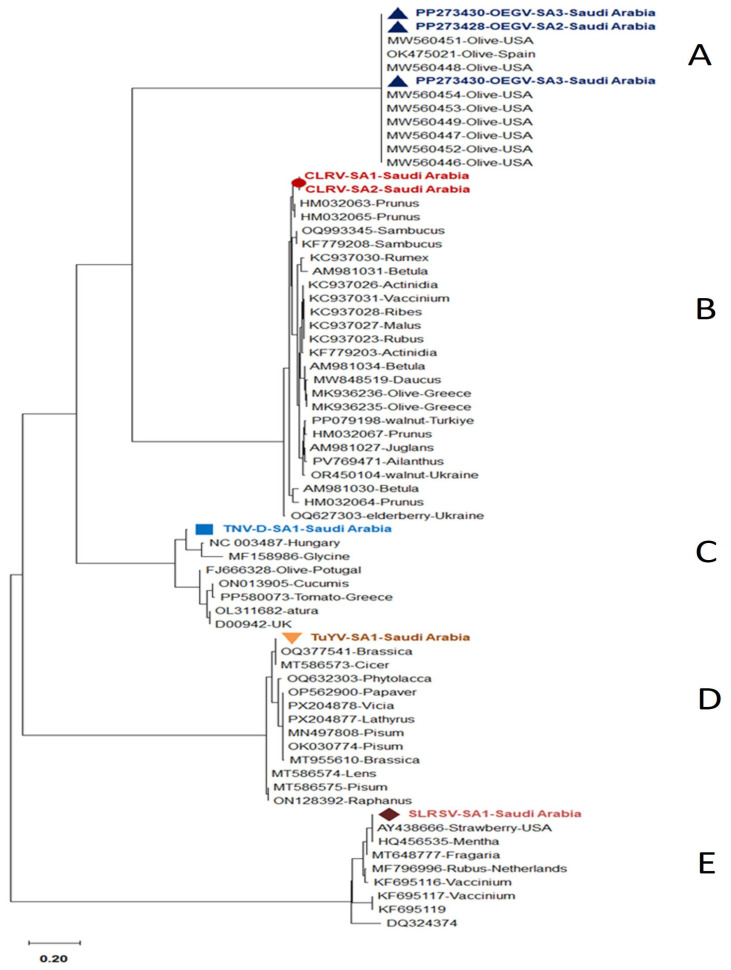
Phylogenetic tree using MEGA-X using the Maximum-Likelihood approach with 1000 bootstrap replications as implemented by MEGA 7.0, based on the alignment of three OEGV (**A**), two CLRV (**B**), and one of each TNV-D (**C**), TuYV (**D**), and SLRSV (**E**) Saudi Arabian isolates sequence with closely related sequences.

**Figure 6:**
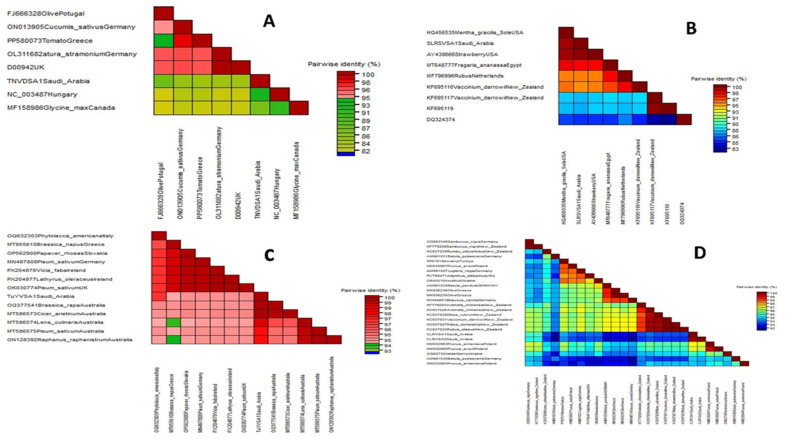
Distance matrix illustrating the pairwise nucleotide identity percentage of one of each TNV-D (**A**), SLRSV (**B**), TuYV (**C**), and two CLRV (**D**) Saudi Arabian isolates as compared with other most similar sequences.

The nucleotide sequence percentage identity between TNV-D isolates showed that our isolates are 84–98% similar to each other. The highest identity (93%) was observed with one isolate from Hungary (NC_003487), and the lowest similarity (84%) was observed with two isolates isolated from *Cucumis sativus* and *Datura stramonium* from Germany (ON013905; OL311682), and one isolate isolated from tomato from Greece and one from the UK (D00942) (Figure 5 and Figure 6A).

The nucleotide sequence percentage identity between SLRSV isolates showed that our isolate is 85–97% similar to each other. The highest identity (97%) was observed with one isolate from Strawberry-USA (AY438666), and the lowest similarity (85%) was observed with one isolate from (DQ324374) (Figure 5 and Figure 6B).

For TuYV isolates, the highest identity (99%) was observed with two isolates isolated from *Brassica rapa* and *Cicer arietinum* in Australia (OQ377541 and MT586573), and the lowest similarity (95%) was observed with one isolate isolated from *Brassica napus* in Greece (MT955610) (Figure 5 and Figure 6C).

For CLRV isolates, the nucleotide sequence percentage identity showed that our isolate is 92–97% similar to each other. The lowest identity (92%) was observed with three isolates isolated from Betula pubescens, Betula pendula, and Daucus carota in Germany (AM981031, AM981034, and MW848519), and the highest similarity (97%) was observed with two isolates isolated from Sambucus nigra in Germany and New Zealand (OQ993345 and KF779208) (Figure 5 and Figure 6D).

**Figure 7:**
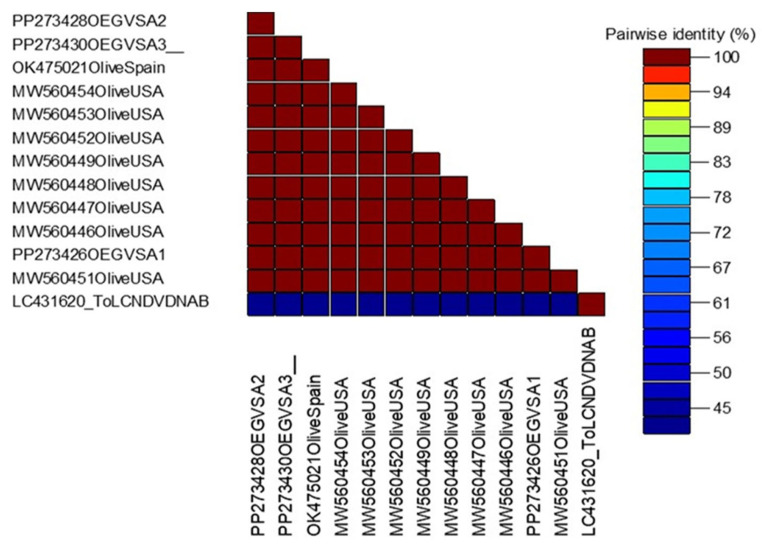
Distance matrix illustrating the pairwise nucleotide identity percentage of three Saudi Arabian isolates of OEGV as compared with other most similar sequences.

Analysis of the sequencing data of OEGV showed that each of the three samples showed 99.7–100% similarity with OEGV isolates. Nucleotide percentage identity showed that our isolates are 100% similar to each other. The highest identity (100%) was observed with all selected NCBI isolates, and the lowest similarity (99.7%) was observed with only one USA isolate (MW460451). Our findings revealed that ten viruses are infecting olive trees in different regions of Saudi Arabia (Figure 5 and Figure 7).

## 4. Discussion

Olives are susceptible to several infections, including bacteria, viruses, fungi, and phytoplasmas [45], which spread through host propagation materials. A few diseases that have been identified affecting olive trees in various areas are probably brought on by viral agents. Fifteen viruses have been reported in several countries, which can infect olive trees, but later on, several viruses were mechanically introduced in the experiment to model plants to study the infectivity and host range; some of them were subsequently analyzed [8,46,47].

Olive-associated viruses are the “ghosts in the machine”—often invisible, but deeply influential. While many of these viruses, such as Olive leaf yellowing-associated virus (OLYaV) or various Olive latent viruses (OLV-1, OLV-2), frequently remain asymptomatic, their importance lies in their ability to quietly compromise yield efficiency and oil chemical composition. Even without killing the tree, a viral load can subtly degrade the sensory qualities of the oil, turning a premium harvest into a mediocre one [48]. According to Zhao et al. (2016) [49], virus-infected plants typically display morphological and physiological changes, which typically lead to decreased production performance. The primary signs of a virus on olive trees are vein clearing, streaking, uneven fruit, and yellowing of the leaves [4]. But some viruses, like OLYaV, can only cause symptoms in some olive cultivars [49]. It is widely recognized that it is difficult to link viral infections to field symptoms in olives [47]. As a result, it is impossible to choose propagation materials without laboratory analyses [5].

The lack of disease symptoms studies in woody experimental plants in biological assays due to slow growth of plants, lower titre of viruses, and the unreliability of the ELISA assay due to lower sensitivity as compared to PCR, makes it difficult to identify virus-infected olive trees. Attempts to perform ELISA for industrial-scale diagnosis of olive trees have actually frequently failed because the extracted sap from olive leaf samples contains tannins and oxidants that can hinder ELISA and reduce its efficiency [50]. However, certain publications on the use of ELISA for SLRSV detection were released [15].

Compared to more conventional detection approaches, the use of molecular diagnostic tools for virus detection has seemed more promising in recent years. Several molecular methods, such as multiplex one-step RT-PCR, nested-RT-PCR, and one-step RT-PCR, have been established based on these principles [6,19,38,39,41,51]. The dependability of these methods encouraged more research on olive virus spread by surveys in various regions [39,52,53].

The purpose of this study was to identify and understand the high viral occurrence in Saudi Arabian olive trees. The outcome of this research indicated the prevalence of ten viruses associated with olive trees in different regions of Saudi Arabia. The results obtained indicated that a total of seven viruses have been detected, including: OEGV, ArMV, SLRSV, CLRV, TuYV, TNV-D, and OMMV in 44.4% of the collected samples with at least one of the tested viruses. The percentage of olive infected samples for each detected virus is as follows: OEGV (14.3%), followed by ArMV (11.9%), SLRSV (11.3%), CLRV (9.4%), TuYV (5%), TNV-D (4.4%), and OMMV (3.6%). OLV-1, OLV-2, CMV, TMV, OLV-3, OLYaV, and OLRSV were found to be negative in the tested samples.

Several studies have been conducted to detect the occurrence of olive viruses in different countries using serological and molecular analysis in Lebanon, with the average infection was 34%, and OLYaV (23.7%) was the most dominant virus, followed by OLV-1, CLRV, ArMV, and the SLRSV [53]. In Tunisia, two studies were conducted on olive viruses in two different years, which indicated the presence of OLYaV, OLV-1, CMV, OLRSV, CLRV, SLRSV, OLV-2, TNV-D, and OMMV [54]. Moreover, in Egypt, CMV (24.7%) was the most common in infected olive trees, followed by OLRSV, OLV-1, CLRV, OLV-2, SLRSV, OLYaV, and ArMV [55]. Similarly, in Syria, CMV was the most prevalent virus, followed by OLRSV, CLRV, and OLYaV [52]. In Morocco, the most prevalent virus was OLYaV (16.2%), followed by the OLV-1, OLV-2, CMV, SLRSV, and ArMV [40].

Furthermore, in Italy, three viruses included OLYaV, SLRSV, and CLRV, have been reported in Southern, Central, and Latium regions, respectively. On average, 32.8% of olive trees were infected with viruses [39]. While in California, OLYaV (93.8%) was the most common virus with the highest infection rate, followed by CMV (34.7%) [56].

A crucial aspect of managing viral diseases is the cultivation of virus-free plant materials, due to the unavailability of scientific information regarding genetic resistance against olive viruses. Phytosanitary certification and selection programs are the primary means of obtaining, propagating, and commercializing plants free of dangerous diseases. Sanitation techniques, including meristem tip culture, micrografting, and heat therapy, can be used to obtain pathogen-free material from sick trees, but their application for virus removal in olives is currently restricted [4]. Therefore, it is crucial to have sensitive, reliable, and economical assays for the identification of olive viruses to ensure sustainable production. Also, the knowledge about the vectors, the potential alternative host (mostly vegetable crops), and the planting method that are involved in the transmission of olive viruses is very important to minimize the infection rate.

However, these viruses were analyzed by PCR, RT-PCR amplification and partial nucleotide sequence. Phylogenetic and DST analyses were carried out to investigate evolutionary relationships among the virus isolates. The partial sequences of ArMV, OMMV, TNV-D, SLRSV, TuYV, and CLRV from olives, which are reported for the first time, showed 83–99% nucleotide similarity with available homologous sequences from other crops, whereas OEGV isolates showed high sequence variability of 99.7–100%. The phylogenetic analysis based on olive detected viruses based on partial-nucleotide sequences grouped each olive virus isolates sequences according to the geographical origins.

The combination of agronomic, ecological, and epidemiological factors could be responsible for the observed regional variations in viral prevalence in Saudi Arabia. Virus introduction and spread can be significantly impacted by differences in olive cultivar composition, tree age, and orchard management techniques, such as the use of uncertified planting material, the severity of pruning, and hygienic standards. Virus stability, host vulnerability, and vector activity can all be impacted by ecological conditions, such as temperature extremes, humidity, and local microclimates. Uneven virus distribution is also caused by epidemiological factors such as variability in nursery sources, plant material transportation between regions, and the presence or lack of potential vectors in a particular region. These elements work together to influence the regional trends in viral prevalence in Saudi Arabia.

This is the first official report of the occurrence of olive viruses in Saudi Arabia, emphasizing the need to implement a certification programme for the production and distribution of high-quality (virus-free) olive propagation material in Saudi Arabia and more generally in the Mediterranean basin.

## 5. Conclusions

The findings indicate a high prevalence of viral infections, with approximately 44.4% of the sampled trees testing positive for at least one virus. Among the viruses detected, OEGV is the most common, affecting nearly 14.3% of the samples. ArMV, SLRSV, and CLRV also showed notable prevalence, while other viruses such as TuYV, TNV-D, and OMMV were present at lower rates. Interestingly, OLV-1, OLV-2, CMV, TMV, OLV-3, OLYaV, and OLRSV were not found in the tested samples. The results provide information about the prevalence and distribution of olive viruses as well as helpful guidance for implementing preventative measures, like sanitary sanitation and selection, which are probably the only practical way to stop the spread of these infections. Several techniques, including high-throughput sequencing (HTS) and full genome sequencing, followed by virus-specific PCR (including RT-PCR and real-time PCR) for confirmation and prevalence investigations, are still required for the detection and confirmation of other olive viruses. Although infections may stay latent, grafting experiments can also verify whether the detected agent is a transmissible virus. Overall, the current findings offer essential baseline information that facilitates the adoption of integrated disease management programs and virus-free olive certification systems, which are crucial for ensuring the sustainability and growth of olive farming in Saudi Arabia. But further studies with full genomes of viruses and their detailed genomic and molecular characterizations are needed to make the olive virome picture clearer.

## Figures and Tables

**Table 1:** A specific set of primers used to detect viruses that infect olive plants.

Virus Name	Forward/ Reverse	Sequence: 5′-3′	Product Size (bp)	Reference
OLV-1	F	tagttaagtatacgaataaca	1200	[38]
	R	aatctggtgttgggtccact		
OLV-2	F	gccaggagtttgagctttg	206	[25,39,40]
	R	gaaggtggctcgcctagag		
OLRSV	F	ctgcaaaactagtgccagagg	492	
	R	tgcataaggctcacaggag		
OLYaV	F	cgaagagagcggctgaaggctc	346	
	R	gggacggttacggtcgagagg		
CMV	F	actcttaaccacccaacctt	280	
	R	aacatagcagagatggcgg		
OLV-3	F-CN10F-M	aattctaccggccaacacct	659	[6]
	R-OLV3-SR	gagggccggaatctgagt		
TMV	TMV5	cgacatcagccgatgcagc	880	[41]
	TMV3	accgttttcgaaccgagact		
TuYV	CP-FP	gttaatgaatacggtcgtgggtag	581	[42]
	CP-RP	attctgaaagaaccagctatcgatg		
ArMV	CP-F	ttggttagtgaatggaacgg	504	[38,40]
	CP-R	tcaactcaccctccaaatccc		
CLRV	conserved region-F	ttggcgaccgtgtaacggca	431	
	conserved region-R	gtcggaaagattacgtaaaagg		
TNV-D	UTR-TNVDd5	gtaggtgacaaggacgctga	278	
	UTR-TNVDd3	ggatagcgactttttagccgct		
OMMV	CP-F	gaatgtctggcgttaagcg	797	[13]
	CP-R	gtgtcctgcgcatcatacac		
SLRSV	SLRSV -F	cctctccaacctgctagact	497	[43]
	SLRSV -R	aagcgcatgaaggtgtaact		
OEGV	OEGV-CP-F	atggattcgtcttccaagaagag	760	This work
	OEGV-P-R	ttaaacacttctgaaccacattcg		

**Table 2:** Total infected samples of infection for each detected olive virus in all main regions in Saudi Arabia using ELISA and PCR.

Region	Tested Samples	Infected Samples	Detected Viruses						
			OEGV	ArMV	SLRSV	CLRV	TuYV	TNV-D	OMMV
Al Jowf	95	43	25	17	12	15	13	12	0
Tabuk	88	42	14	14	21	9	0	0	12
Hail	77	63	13	7	1	7	2	0	1
Qassim	35	3	0	1	0	1	0	0	0
Abha Asser	21	2	0	1	2	1	0	0	0
Northern Borders	20	7	0	2	4	1	3	3	0
Al-Bahah	27	1	0	1	1	0	0	1	0
**Total**	**363**	**161**	**52**	**43**	**41**	**34**	**18**	**16**	**13**

**Table 3:** The percentage of olive-infected samples for each detected virus in each region in all main regions in Saudi Arabia using ELISA and PCR.

Region	Tested Samples	% of Detected Viruses						
		OEGV	ArMV	SLRSV	CLRV	TuYV	TNV-D	OMMV
Al Jowf	95	26.3	17.9	12.6	15.8	13.7	12.6	0
Tabuk	88	15.9	15.9	23.9	10.2	0	0	13.6
Hail	77	16.9	9.1	1.3	9.1	2.6	0	1.3
Qassim	35	0	2.9	0	2.9	0	0	0
Abha Asser	21	0	4.8	9.5	4.8	0	0	0
Northern Borders	20	0	10	20	5	15	15	0
Al-Bahah	27	0	3.7	3.7	0	0	3.7	0
**%**	**-**	**44.4**	**14.3**	**11.9**	**11.3**	**9.4**	**5**	**4.4**

**Table 4:** Distribution of the percentage of single and mixed infection samples in olive trees in Saudi Arabia using ELISA and PCR.

Region	Tested Samples	Infected Samples	% Single Infection	% Mixed Infection
Al Jowf	95	43	9	34
Tabuk	88	42	3	38
Hail	77	63	19	44
Qassim	35	3	2	1
Abha Asser	21	2	0	2
Northern Borders	20	7	1	6
Al-Bahah	27	1	0	1
Total	**363**	**161**	**0**	**0**
**%**	**-**	**44.4**	**9.4%**	**34.7%**

## Data Availability

While more information can be obtained upon request, some data is provided in the article.
